# *In vitro* activity of delafloxacin against enterococcal isolates from male urinary tract infections and genetic determinants of resistance

**DOI:** 10.1128/spectrum.01170-26

**Published:** 2026-08-24

**Authors:** Adrien Turban, Benjamin Quenet, Charlotte Michaux, François Guérin, Vincent Cattoir

**Affiliations:** 1 Department of Bacteriology, University Hospital of Rennes, Rennes, France; 2 Inserm U1230 Bacterial RNAs and Medicine, University of Rennes 27079, Rennes, France; 3 National Reference Center for Enterococci, University Hospital of Rennes 679356, Rennes, France; Quest Diagnostics Nichols Institute, Chantilly, Virginia, USA

**Keywords:** delafloxacin, fluoroquinolones, *E. faecalis*, UTI

## Abstract

Delafloxacin (DLX) is a novel fluoroquinolone (FQ) with enhanced activity against gram-positive bacteria and under acidic conditions. Since the pH of urine is usually acidic, DLX could be an alternative to current drugs for urinary tract infections (UTIs), especially in male patients, for whom good prostatic tissue penetration is required. The aim of this study was to evaluate *in vitro* the activity of DLX and FQ comparators against *Enterococcus faecalis* and *Enterococcus faecium*, to characterize the resistance pattern of *E. faecalis* against FQs, and to decipher the genetic basis of resistance among *in vitro*-selected mutants. DLX showed higher *in vitro* activity than both levofloxacin and moxifloxacin against *E. faecalis*, with an MIC_50_ of 0.06 mg/L, which was 16- and 4-fold lower than those of these two FQs, respectively. None of the three antibiotics was effective against *E. faecium* (MICs mostly >8 mg/L). DLX exhibited increased activity under acidic conditions, as the MIC_50_ for 12 *E. faecalis* isolates decreased by a 3-log_2_ dilution between pH 7.2 and pH 5, whereas comparator activities were impaired. A total of 19 mutants with significant increases in DLX MICs were selected *in vitro*, including 12/19 (63%) with at least one mutation in the quinolone resistance-determining region (QRDR) of GyrA (mainly at the position S84). Interestingly, four mutants lacked QRDR mutations but carried mutations in *eno* or *infB* genes, which have not previously been reported in FQ resistance. In conclusion, DLX showed greater *in vitro* activity than levofloxacin and moxifloxacin against *E. faecalis* isolates, especially under acidic conditions, suggesting its potential use for treating male UTIs.

**IMPORTANCE:**

Enterococci urinary tract infections (UTIs) are common but often difficult to treat in male patients, in whom good prostatic tissue penetration is required. Fluoroquinolones (FQs) remain attractive in this setting even though their efficacy is altered by the acidic pH of urine. Delafloxacin (DLX) is a novel FQ whose structure confers enhanced activity against gram-positive bacteria and under acidic conditions such as urine. Here, we demonstrate that DLX is substantially more active *in vitro* against *Enterococcus faecalis* than levofloxacin or moxifloxacin, an advantage further amplified under urine-mimicking acidic conditions. The low frequency of *in vitro* spontaneous resistant mutants and the predominance of GyrA substitutions among mutants suggest a resistance driven by target modification. Together, these results identify DLX as a promising candidate to address an unmet need in the management of male enterococcal UTIs, but further validation in animal models and clinical studies is needed.

## INTRODUCTION

Urinary tract infections (UTIs) are among the most common bacterial infections, affecting approximately 400 million people every year (1). Although UTIs predominantly affect women, around 20% of men will experience at least one episode during their lifetime (2, 3). Enterococci are among the leading uropathogens in men, with *Enterococcus faecalis* being the second most frequently isolated species (13.2%) after *Escherichia coli* (3). Diagnosis of male UTIs remains challenging, as concomitant prostatitis can rarely be excluded (2, 4, 5); antibiotic therapy must therefore rely on agents with high diffusion into prostatic tissue, such as fluoroquinolones (FQs) (4, 5). For male UTIs due to *E. faecalis*, β-lactams—mainly ampicillin or amoxicillin—are recommended as first-line agents, leaving few validated options for penicillin-allergic patients (4). Another obstacle to effective therapy is the acidic microenvironment of infected urine: indeed, during a UTI, urine pH commonly falls below 7 in approximately 90% of cases (6, 7). Most conventional FQs, which exist as zwitterions at physiological pH, lose antibacterial potency under such acidic conditions, with their MICs rising as pH decreases (8–11).

In this context, delafloxacin (DLX; formerly WQ-3034, ABT-492) may represent an attractive candidate, particularly since reported FQ resistance among *E. faecalis* causing these infections remains low (4.9%) (3). DLX is the only anionic (non-zwitterionic) FQ in clinical use: the lack of a basic substituent at C7 makes it a weak acid (pKa ≈ 5.5), predominantly anionic at neutral pH but mostly uncharged at acidic pH (≤5.5) (10, 12, 13). The neutral form crosses bacterial membranes more readily, leading to high intracellular accumulation and enhanced activity under acidic conditions. Accordingly, DLX MICs significantly decrease as pH falls from 7.2 to 5.5, with the most pronounced effects against gram-positive bacteria (7, 9–11). DLX also inhibits both type II topoisomerases (i.e., DNA gyrase and topoisomerase IV) with nearly balanced affinity, a feature expected to limit resistance selection, as this would require mutations to accumulate in both targets (11, 13–15). Its *in vitro* activity against enterococci, however, has been only sparsely documented. DLX is consistently active against *E. faecalis* (MIC_50_ and MIC_90_ at 0.06–0.12 and 1 mg/L, respectively), whereas it is essentially inactive against *Enterococcus faecium* (MIC_90_ >4 mg/L) (11, 12, 15–17). Resistance to FQs in enterococci arises primarily through mutations in the quinolone-resistance-determining regions of *gyrA* (DNA gyrase) and *parC* (topoisomerase IV)—the latter being the preferential target in gram-positive bacteria—together with efflux mechanisms (10, 14, 18, 19).

The aim of this study was therefore to compare the *in vitro* activity of DLX with that of FQ comparators (i.e., levofloxacin [LVX] and moxifloxacin [MOX]) by determining minimal inhibitory concentration (MIC) values under neutral and acidic conditions and minimal bactericidal concentrations (MBCs) against a large collection of enterococcal isolates responsible for male UTIs. We further characterized the resistance patterns of *E. faecalis* to FQs by determining mutant prevention concentration (MPC) values and frequencies of spontaneous mutant selection (FSMS) and deciphered the genetic basis of resistance through comparative genomic analysis between *in vitro*-selected mutants and their parental strains.

## RESULTS

### Comparative *in vitro* activity

Because enterococci are intrinsically less susceptible to FQs than gram-negative uropathogens, we first asked whether DLX was more active than the comparators LVX and MOX against urinary *E. faecalis*, given its unique structural features (including the absence of a basic group at C7, the presence of a chlorine substituent at C8, and a heteroaromatic group at N1) contributing to its enhanced antibacterial potency (10, 12). MIC and MBC values were therefore determined for the three FQs in cation-adjusted Mueller–Hinton II broth (CA-MHB). MICs for 86 *E. faecalis* isolates ranged from 0.5 to >8 mg/L for LVX, from 0.125 to >8 mg/L for MOX, and from 0.03 to 1 mg/L for DLX (Table 1 and Table S1). DLX showed greater *in vitro* activity than LVX and MOX against *E. faecalis*, with MIC_50_ (0.06 mg/L) and MIC_90_ (0.25 mg/L) values that were 16- and ≥32-fold lower than those obtained for LVX, and 4- and 32-fold lower than those for MOX, respectively (Table 1). According to 2026 CLSI or FDA breakpoints, 14%, 13%, and 8% of *E. faecalis* isolates were resistant to LVX, MOX, and DLX, respectively (Table 1). Against the 12 norfloxacin (NFX)-resistant *E. faecalis* isolates, DLX retained markedly lower MIC_50_ and MIC_90_ values (0.5 and 1 mg/L, respectively) than LVX (both >8 mg/L) and MOX (both >8 mg/L). Consequently, only 58% of these isolates were categorized as resistant to DLX compared with 100% and 92% for LVX and MOX, respectively (Table 1). Bactericidal activity, defined as an MBC/MIC ratio ≤4, was observed in 71/74 (96%), 71/74 (96%), and 72/74 (97%) of the NFX-susceptible *E. faecalis* strains for LVX, MOX, and DLX, respectively (Table 1 and Table S1). Interestingly, DLX kept a bactericidal activity against all the 12 NFX-resistant strains (Table S1). All of the 24 *E. faecium* isolates were highly resistant to the three tested FQs (Table 1 and Table S2). Overall, DLX displayed higher *in vitro* activity than LVX and MOX against *E. faecalis*, with the difference being most pronounced among NFX-susceptible isolates. Against NFX-resistant isolates, MICs were increased, but DLX retained substantially lower MICs than both comparators and remained bactericidal against all of them.

**TABLE 1:** MIC and MBC values (mg/L) of delafloxacin and comparators against *E. faecalis* and *E. faecium* clinical isolates at pH 7.2^*d*^

Strain	No. of isolates	MIC^*a*^ (mg/L)				MBC^*a*^^,^^*b*^ (mg/L)				MBC/MIC^*a*^^,*b*^		
		Results	LVX	MOX	DLX	Results	LVX	MOX	DLX	LVX	MOX	DLX
*E. faecalis*	86	MIC_50_	1	0.25	0.06	MBC_50_	–	–	0.125	–	–	2
		MIC_90_	>8	8	0.25	MBC_90_	–	–	0.5	–	–	2
		Range	0.5 to >8	0.125 to >8	0.03 to 1	Range	–	–	0.03 to 2	–	–	–
		Resistance (%)^*c*^	14	13	8							
*E. faecalis* NFX-S	74	MIC_50_	1	0.25	0.06	MBC_50_	1	0.25	0.125	1	1	2
		MIC_90_	2	0.5	0.125	MBC_90_	2	0.5	0.125	1	1	1
		Range	0.5 to 2	0.125 to 0.5	0.03 to 0.125	Range	1 to >8	0.125 to 2	0.03 to 1			
		Resistance (%)^*c*^	0	0	0							
*E. faecalis* NFX-R	12	MIC_50_	>8	>8	0.5	MBC_50_	–	–	0.5	–	–	1
		MIC_90_	>8	>8	1	MBC_90_	–	–	2	–	–	2
		Range	>8	2 to >8	0.125 to 1	Range	–	–	0.125 to 2	–	–	–
		Resistance (%)^*c*^	100	92	58							
*E. faecium*	24	MIC_50_	>8	>8	>8	MBC_50_	–	–	–	–	–	–
		MIC_90_	>8	>8	>8	MBC_90_	–	–	–	–	–	–
		Range	>8	>8	2 to >8	Range	–	–	–	–	–	–
		Resistance (%)^*c*^	100	100	100							

^*a*^ Individual MIC, MBC, and MBC/MIC values are reported in Tables S1 and S2.

^*b*^ MBC values for LVX and MOX were determined only for NFX-S isolates.

^*c*^ According to 2026 CLSI breakpoint for LVX (resistant if >4 mg/L) and 2026 FDA breakpoints for MOX and DLX (resistant if >2 or >0.25 mg/L, respectively).

^*d*^ –, not determined; DLX, delafloxacin; LVX, levofloxacin; MOX, moxifloxacin; NFX-R, norfloxacin resistant; NFX-S, norfloxacin susceptible.

### DLX *in vitro* activity under acidic conditions

**Fig 1:**
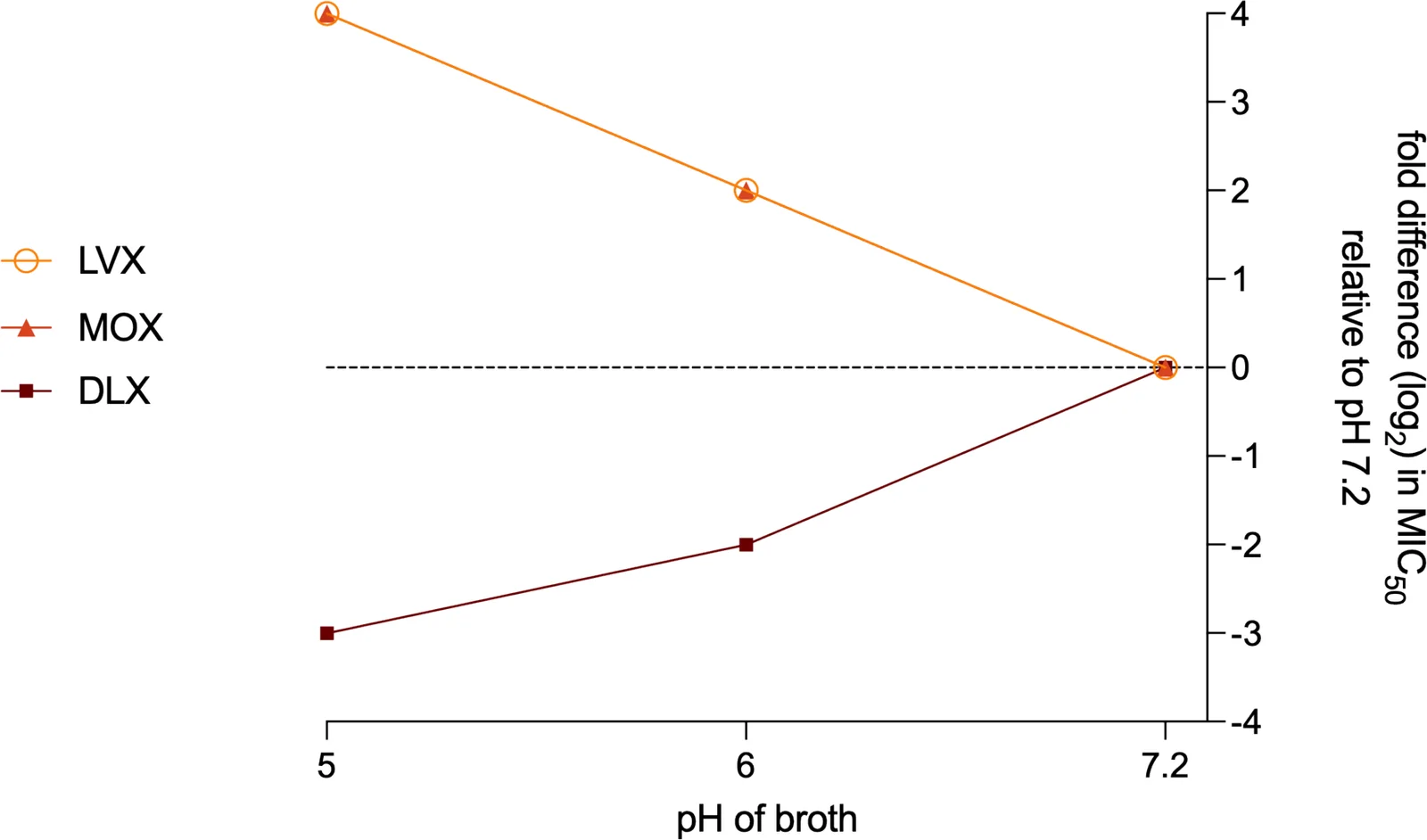
Evolution of levofloxacin, moxifloxacin, and delafloxacin MIC_50_ against *E. faecalis* clinical isolates relative to pH. LVX, levofloxacin; MOX, moxifloxacin; DLX, delafloxacin. Data points represent log_2_ fold differences in MIC_50_ relative to pH 7.2; individual MIC values are provided in Table S3.

**Fig 2:**
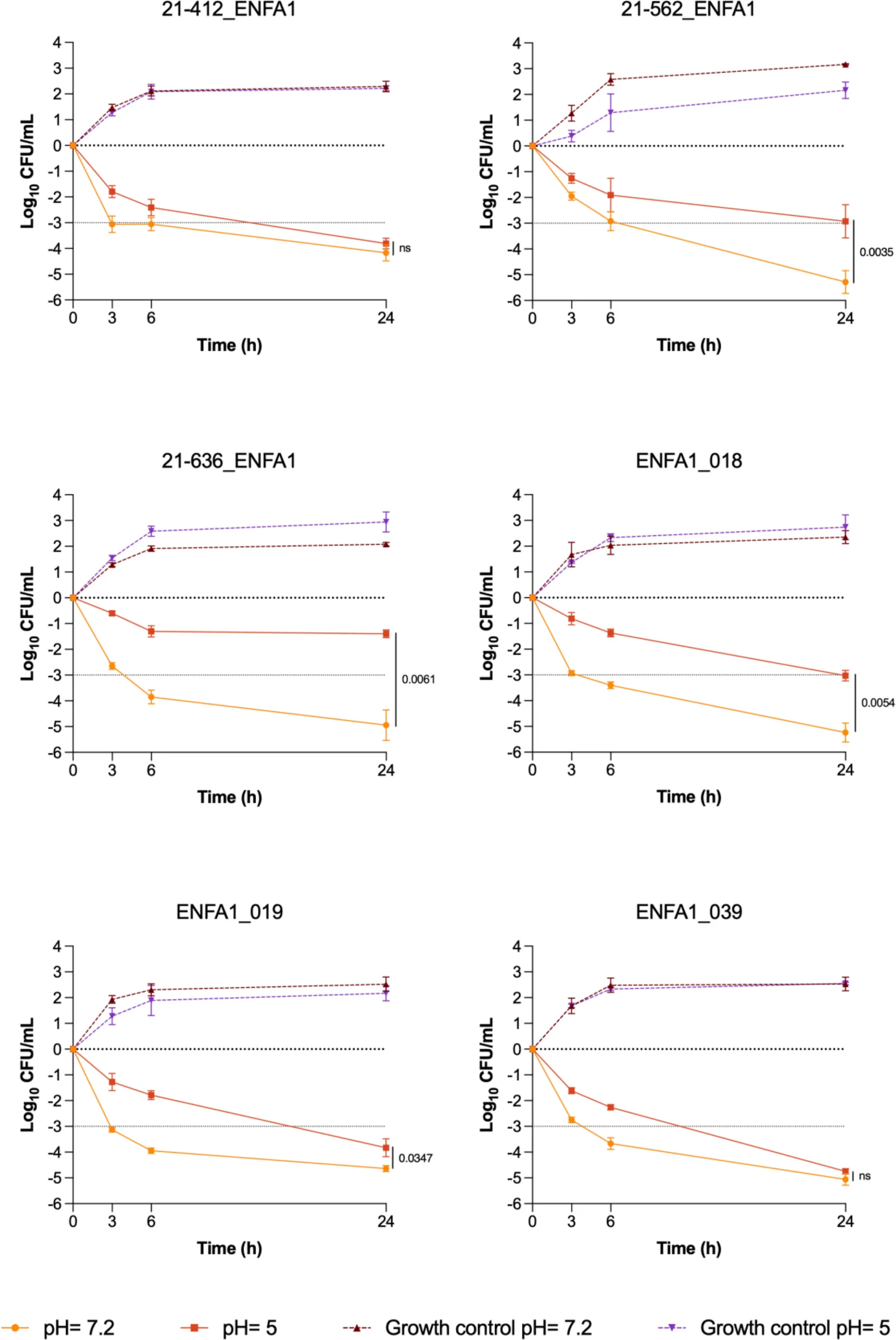
Mean time-kill curves of delafloxacin (50 × MIC) for six *E. faecalis* strains. Reduction of bacterial count is expressed as the log_10_ of the ratio between the inoculum at T3, T6, or T24 and T0 time points. *P* values are indicated (paired *t*-test on log_10_-transformed T24/T0 ratios); error bars represent the mean ± SD of triplicate experiments.

Having established the superior potency of DLX at neutral pH, we next asked whether this advantage is maintained—or improved—under acidic conditions that characterize urine, since the anionic, non-zwitterionic nature of DLX predicts a pH-dependent behavior distinct from that of conventional FQs. To better reflect UTI pathophysiology, MIC values of LVX, MOX, and DLX were determined under urinary-mimicking acidic conditions (acidic CA-MHB [aCA-MHB]) using a panel of 12 *E. faecalis* strains representing the overall distribution of DLX MICs. DLX exhibited increased *in vitro* activity under acidic conditions, as the MIC_50_ for the 12 *E. faecalis* isolates decreased by a 3-log_2_ dilution between pH 7.2 and pH 5 (Table 2; Table S3; Fig. 1). Both comparators showed an opposite pattern through the same pH range, as MIC_50_ for LVX and MOX increased by 4-log_2_ dilutions (Table 2; Table S3; Fig. 1). Surprisingly, although DLX showed decreased MICs at pH 5, several isolates showed increased MBC/MIC ratios (>4 for 7/12, 58%) with no clear explanation (Table S4). Then, *in vitro* bactericidal activity of DLX was studied through time-kill (TK) assay under two pH conditions (pH 7.2 and 5). Six isolates (including two with MBC/MIC ratio <4) were tested in this dynamic model, and the logarithmic reductions of bacterial cells (log_10_ CFU/mL) are presented in Fig. 2. DLX exhibited bactericidal activity against all six *E. faecalis* isolates at 24 h in neutral brain-heart infusion (nBHI) (Fig. 2; Table S5). Under acidic conditions, four of them achieved a 3-log_10_ CFU/mL reduction at 24 h, and strain 21-562_ENFA1 showed a nearly bactericidal 2.92-log_10_ CFU/mL reduction (Fig. 2; Table S5), although its MBC/MIC ratio in aCA-MHB was <4 (Table S4). Although four isolates had MBC/MIC ratios up to >64 at pH 5 (Table S4), only one of these strains, 21-636_ENFA1, failed to achieve a 3-log10 CFU/mL reduction from the initial bacterial inoculum after 24 h in acidic BHI (aBHI), with a mean reduction of 1.40 log_10_ CFU/mL (Fig. 2; Tables S4 and S5). Among the four strains against which DLX exerted a ≥3-log_10_ CFU/mL reduction under acidic conditions, bactericidal activity of DLX was achieved earlier at pH 7.2 than at pH 5 for each strain (Fig. 2; Table S5). Indeed, under the neutral condition, a 3-log_10_ CFU/mL reduction was observed for all of the four strains as early as 6 h, whereas at pH 5, none of the isolates showed such reduction (Fig. 2; Table S5). In summary, DLX exerted bactericidal activity in TK experiments against 6/6 strains under neutral conditions and 4/6 strains under acidic conditions. Among the four strains for which DLX retained bactericidal activity, two showed significantly lower bacterial killing at 24 h under acidic compared with neutral conditions.

**TABLE 2:** MIC values (mg/L) of delafloxacin and comparators against 12 *E. faecalis* clinical isolates at different pH values^*c*^

	LVX^*a*^			MOX^*a*^			DLX^*a*^		
	MIC_50_	MIC_90_	Range	MIC_50_	MIC_90_	Range	MIC_50_	MIC_90_	Range
pH 7.2	1	>8	0.5 to >16	0.25	>8	0.125 to >8	0.06	1	0.03 to 1
pH 6	4	>16	2 to >16	1	>16	0.25 to >16	0.016	0.125	0.004 to 0.25
pH 5	16	>16	4 to >16	4	>16	1 to >16	0.008	0.25	0.001 to 0.25
Log_2_ fold changes in MIC (pH 6)^*b*^	2	–	–	2	–	–	−2	−3	–
Log_2_ fold changes in MIC (pH 5)^*b*^	4	–	–	4	–	–	−3	−2	–

^*a*^ Individual MIC values are provided in Table S3.

^*b*^ values are expressed as log_2_ fold differences relative to pH 7.2.

^*c*^ –, not determined; DLX, delafloxacin; LVX, levofloxacin; MOX, moxifloxacin.

### *In vitro* selection of spontaneous resistant mutants

Beyond intrinsic activity, the clinical value of an FQ also depends on how readily it selects for resistance. We therefore determined FSMS and MPC of the three FQs, testing the hypothesis that the comparable affinity of DLX for both DNA gyrase and topoisomerase IV (15) might limit the *in vitro* selection of first-step resistant mutants. For FSMS and MPC determination, we used six different *E. faecalis* strains at 2-, 4-, 8-, and 16-fold the MIC, with a maximum concentration of 32 mg/L for LVX and MOX in BHI agar plates. As a dense lawn or uncountable number of bacterial cells were frequently encountered at high bacterial concentration (~10^9^ CFU), a lower inoculum was subsequently used (~10^7^ CFU), and the highest FSMS value across both inocula and replicates measurements was reported in Table 3. At 2-fold the MIC, the median FSMS was 5.55 × 10^−8^, 5.10 × 10^−8^, and 6 × 10^−9^ for LVX, MOX, and DLX, respectively. At a concentration of 4-fold the MIC, median FSMS values were 4 × 10^−9^ for MOX and <1 × 10^−9^ for both LVX and DLX. At higher concentrations, the median FSMS was <1 × 10^−9^ for all three tested molecules.

**TABLE 3:** MPC and FSMS values for six *E. faecalis* clinical isolates^*c*^

Strain		Agent tested	MIC (mg/L)	MPC (mg/L)	MSW (mg/L)	SI^*a*^	FSMS^*b*^			
							2 × MIC	4 × MIC	8 × MIC	16 × MIC
21-562_ENFA1		LVX	2	4	2–4	2	1 × 10^−9^	<1 × 10^−9^	<1 × 10^−9^	<1 × 10^−9^
		MOX	0.5	1	0.5–1	2	<1 × 10^−9^	<1 × 10^−9^	<1 × 10^−9^	<1 × 10^−9^
		DLX	0.125	0.25	0.125–0.25	2	<1 × 10^−9^	<1 × 10^−9^	<1 × 10^−9^	<1 × 10^−9^
ENFA1_002		LVX	1	4	1–4	4	3 × 10^−7^	<1 × 10^−9^	<1 × 10^−9^	<1 × 10^−9^
		MOX	0.25	>4	0.25–>4	>16	<1 × 10^−9^	3 × 10^−9^	5 × 10^−9^	1 × 10^−9^
		DLX	0.06	0.125	0.06–0.125	2	<1 × 10^−9^	<1 × 10^−9^	<1 × 10^−9^	<1 × 10^−9^
ENFA1_003		LVX	2	4	2–4	2	<1 × 10^−9^	<1 × 10^−9^	<1 × 10^−9^	<1 × 10^−9^
		MOX	0.5	1	0.5–1	2	2 × 10^−9^	5 × 10^−9^	<1 × 10^−9^	<1 × 10^−9^
		DLX	0.125	0.25	0.125–0.25	2	1 × 10^−9^	<1 × 10^−9^	<1 × 10^−9^	<1 × 10^−9^
ENFA1_004		LVX	1	4	1–4	4	1.1 × 10^−8^	<1 × 10^−9^	<1 × 10^−9^	<1 × 10^−9^
		MOX	0.125	>2	0.125–>2	>16	2.7 × 10^−6^	2 × 10^−7^	7 × 10^−9^	1 × 10^−9^
		DLX	0.03	0.125	0.03–0.125	4	2.5 × 10^−6^	6 × 10^−9^	<1 × 10^−9^	<1 × 10^−9^
ENFA1_006		LVX	1	4	1–4	4	1 × 10^−7^	<1 × 10^−9^	<1 × 10^−9^	<1 × 10^−9^
		MOX	0.25	1	0.25–1	4	1 × 10^−7^	9 × 10^−9^	<1 × 10^−9^	<1 × 10^−9^
		DLX	0.06	0.25	0.06–0.25	4	1.8 × 10^−8^	<1 × 10^−9^	<1 × 10^−9^	<1 × 10^−9^
ENFA1_019		LVX	0.5	2	0.5–2	4	3.5 × 10^−6^	<1 × 10^−9^	<1 × 10^−9^	<1 × 10^−9^
		MOX	0.125	1	0.125–1	8	6 × 10^−7^	2 × 10^−9^	1 × 10^−9^	<1 × 10^−9^
		DLX	0.03	0.06	0.03–0.06	2	1.1 × 10^−8^	<1 × 10^−9^	<1 × 10^−9^	<1 ×10^−9^

^*a*^ SI is expressed as the ratio between MPC/MIC.

^*b*^ The highest FSMS value across both inocula and replicate measurements is presented.

^*c*^ DLX, delafloxacin; FSMS, frequency of spontaneous mutant selection; LVX, levofloxacin; MOX, moxifloxacin; MPC, mutant prevention concentration; MSW, mutant selection window; SI, selection index.

Using BHI agar plates, MPC values ranged from 2 to 4 mg/L, 1 to >4 mg/L, and from 0.06 to 0.25 mg/L for LVX, MOX, and DLX, respectively (Table 3). We then determined the mutant selection window (MSW), which represents the concentration range in which resistant mutant isolates are more likely to emerge (i.e., [MIC-MPC] window), as well as the ratio MPC/MIC (also referred to as selection index [SI]) (20–22). The SI values ranged from 2 to 4, 2 to >16, and 2 to 4 for LVX, MOX, and DLX, respectively (Table 3). Taken together, the data were comparable between DLX and LVX, although FSMS for DLX were lower at 2-fold the MIC. In contrast, MOX exhibited the highest FSMS and MPC values among the three tested molecules in this study.

### Genetic basis of DLX resistance in *E. faecalis*

To uncover the molecular determinants underlying the resistance selected *in vitro*, the parental strains and a selection of their derived mutants were subjected to whole-genome sequencing (WGS) and comparative genomic analysis. Sequence types (STs), determined from WGS data, were used to describe the diversity of genetic backgrounds of parental isolates and indicate that resistant mutants could emerge from diverse lineages. A total of eight *E. faecalis* parental strains, belonging to different STs (ST16, ST25, ST34, two ST40, ST81, ST179, and ST480), were used, and *in vitro* resistant mutants were selected for each of them (Table 4). A total of 22 mutants with significant increase in MICs (>1 two-fold dilution) for at least one of the FQs tested were selected based on their DLX MICs to ensure a representative range of susceptibilities. By comparison with the reference strain ATCC 29212 (ST30), quinolone resistance-determining region (QRDR) mutations were detected in 2/8 (25%) of the parental isolates (Table 4). Strain 21-517_ENFA1 harbored mutations in both GyrA (E88G, *E. faecalis* numbering) and ParC (S82I), which were associated with resistance to LVX. Furthermore, strain 21-439_ENFA1, that carried the same mutation in ParC, in combination with a different substitution in GyrA (S84Y), showed resistance to the three FQs.

**TABLE 4:** Genomic characterization of *in vitro*-selected fluoroquinolone-resistant mutants from eight *E. faecalis* clinical isolates^*b*^

Strain	Isolation	MIC (mg/L)			ST	Mutations in FQ targets										MIC changes^*a*^		
		LVX	MOX	DLX		*gyrA*			*gyrB*		*parC*		*parE*			LVX	MOX	DLX
						S	E	D	D	S	S	K	D	P	A			
						84	88	760	437	442	82	460	434	453	656			
ENFA1_019		0.5	0.125	0.03	40							N				–	–	–
Mutant 1	DLX	1	0.25	**0.125**								N				1	1	2
Mutant 2	DLX	**2**	**1**	**0.25**		**I**						N				2	3	3
Mutant 3	MOX	**2**	**1**	0.06					**N**			N				2	3	1
ENFA1_004		1	0.125	0.03	179										T	–	–	–
Mutant 1	DLX	2	**1**	**0.25**		**I**									T	1	3	3
Mutant 2	LVX	2	0.25	**0.125**											T	1	1	2
Mutant 3	LVX	**4**	**1**	0.06									**N**		T	2	3	1
Mutant 4	MOX	1	**0.5**	**0.125**		**I**									T	0	2	2
Mutant 5	MOX	**4**	**2**	**0.25**		**F**									T	2	4	3
ENFA1_006		1	0.25	0.06	40											–	–	–
Mutant 1	DLX	**4**	**1**	**0.5**												2	2	3
Mutant 2	DLX	**8**	**2**	**0.5**												3	3	3
Mutant 3	LVX	**4**	0.5	**0.25**										S		2	1	2
Mutant 4	MOX	**4**	**1**	**0.25**			**K**									2	2	2
ENFA1_002		1	0.25	0.06	25											–	–	–
Mutant 1	MOX	**4**	**2**	**1**		**I**										2	3	4
Mutant 2	MOX	**4**	**2**	**1**		**R**										2	3	4
ENFA1_003		2	0.5	0.125	34											–	–	–
Mutant 1	DLX	4	**2**	**1**		**I**										1	2	3
Mutant 2	MOX	4	**4**	**0.5**		**R**										1	3	2
21-562_ENFA1		2	0.5	0.125	81											–	–	–
Mutant 1	LVX	4	**2**	**1**												1	2	3
21-517_ENFA1		16	2	0.125	16		**G**				**I**				T	–	–	–
Mutant 1	DLX	**64**	**32**	**4**		**I**	**G**				**I**				T	2	4	5
Mutant 2	MOX	32	**32**	**0.5**		**R**	**G**				**I**				T	1	4	2
Mutant 3	MOX	**64**	**32**	**4**		**I**	**G**				**I**				T	2	4	5
Mutant 4	MOX	32	**8**	0.25		**C**	**G**				**I**				T	1	2	1
21-439_ENFA1		32	8	0.5	480	**Y**		N			**I**				T	–	–	–
Mutant 1	DLX	64	**32**	**4**		**Y**		N		**P**	**I**				T	1	2	3

^*a*^ Change in MIC is expressed as log_2_ fold dilution compared to the related parental strain.

^*b*^ DLX, delafloxacin; LVX, levofloxacin; MOX, moxifloxacin; ST, sequence type. Underlined letters indicate amino acid mutations present in the parental strain compared to the reference strain (ATCC 29212). Non-underlined letters indicate mutations acquired during *in vitro* selection in the mutant compared to the related parental strain. Bold letters indicate mutations occurring within the QRDR. MIC values in bold correspond to mutants with >1 two-fold dilution increase in MIC relative to the parental strain.

Within the selected mutant collection, 12/22 (54%), 19/22 (86%), and 19/22 (86%) of the isolates exhibited a significant increase in MICs of LVX, MOX, and DLX, respectively (Table 4). Our analysis focused on mutations within the QRDRs of *gyrA*, *gyrB*, *parC*, and *parE* genes. Sixteen (73%) isolates acquired QRDR mutations during the *in vitro* selection: 11/16 (69%) showed single substitutions, and 5/16 (31%) harbored three different mutations (Table 4 and Table S6). The most frequent QRDR mutations were found in GyrA (13/16, 81%), with S84I (7/13, 54%) prevailing, followed by S84R (3/13, 23%), and the mutations S84F, S84C, or E88K were found in 1/13 (8%) of isolates (Table 4). Three mutants harbored QRDR mutations within GyrB or ParE, and no mutation was detected within the ParC subunit (Table 4). Among the 19 mutants with significant increases in DLX MICs, 13/19 (68%) carried at least one mutation in QRDRs, whereas 6/19 (32%) did not harbor any mutation in those targets. Among these 13 mutants, 9/13 (69%) and 4/13 (31%) harbored single or triple substitutions, respectively (Table 4). Notably, in all of the nine isolates carrying a single QRDR mutation, amino acid substitutions occurred in GyrA at positions S84 (*n* = 8; 5 S84I, 2 S84R, 1 S84F) or E88 (1 E88K). This unique mutation was responsible for a 4- to 16-fold increase in the DLX MIC (Table 4). The four mutants harboring three QRDR substitutions corresponded to parental strains that already harbored two substitutions in QRDRs of GyrA and ParC. All of them acquired an additional mutation in GyrA (two S84I, one S84R) or GyrB (one S442P), in three and one isolates, respectively, which conferred a 2- to 32-fold increase in the MIC of DLX (Table 4). While all mutants carrying mutations in DNA gyrase subunits (i.e., GyrA or GyrB) were selected on agar plates containing either MOX or DLX, the only mutant harboring a mutation in the QRDR of ParE was selected on an LVX-containing agar plate (Table 4). Out of the six mutants that did not harbor any mutation in QRDRs, three were obtained from the same parental strain (ENFA1_006) (Table S7). Interestingly, substitutions were identified in the same gene, *eno*, coding for phosphopyruvate hydratase, for 3/6 mutants (actually corresponding to two different genetic backgrounds—ENFA1_006 and 21-562_ENFA1), while a mutant from ENFA1_006 harbored a single nonsense mutation in the *infB* gene, coding for the translation initiation factor IF-2 (Table S7). Altogether, these data demonstrate that resistance to DLX in *E. faecalis* mainly relies on QRDR mutations in GyrA, particularly at the S84 position, but that it may be related to mutations in other genes not known to be involved in FQ resistance.

## DISCUSSION

This *in vitro* study highlights several important features about DLX potential use in enterococcal UTIs, especially in male patients. Firstly, DLX was the most active against *E. faecalis* in our collection, with MIC_50_ and MIC_90_ significantly lower than those of LVX and MOX. Secondly, the *in vitro* activity of DLX was increased under urine-mimicking acidic conditions, as the MIC_50_ values were decreased by a 3-log_2_ dilution between pH 7.2 and pH 5. As an anionic, non-zwitterionic agent, DLX mostly exists under non-ionized form in pH <7, which may potentialize its penetration into bacterial cells, as uncharged molecules are more likely to diffuse through cellular membranes, thereby accentuating its antibacterial activity (7, 9, 13, 23). Even though DLX MICs under acidic conditions were decreased, it appears that, for several isolates, bactericidal activity was impaired in this environment, with MBC values higher than those obtained in neutral broth. To further characterize this observation, we used a dynamic *in vitro* model with TK assays, using high concentrations of DLX. Interestingly, we found discrepancies between MBC/MIC ratios and TK assays. Indeed, even though MBC/MIC ratios were >64 for several isolates, TK assays at 50-fold the MIC demonstrated bactericidal activity for the majority of the tested isolates. These discrepant results between MBC and TK experiments in bacterial reduction could be explained by several factors. First, the observed discrepancies may be explained by the inherent variability in MIC and MBC determinations and by differences between MIC measured by the broth microdilution (BMD) method and those obtained under higher-volume conditions, which can substantially alter the MBC/MIC ratio. Second, unlike the MBC determination method, which is an end-point experiment, TK assay is a dynamic model that allows a more precise assessment of the bacterial killing. Third, differences in bacterial growth phase and medium composition may also contribute to these discrepancies. Finally, the high concentration used in TK experiments was chosen to challenge isolates showing high MBC/MIC ratios (>64), for which bactericidal activity would not be expected based on standard interpretation. Also, from an *in vivo* perspective relevant to UTIs, it is worth noting that a substantial fraction (30–45%) of DLX is excreted unchanged in urine (24), so urinary concentrations are expected to markedly exceed the MICs of most *E. faecalis* isolates (MIC_50_ at pH 5 = 0.008 mg/L). Overall, both methods consistently showed that DLX bacterial killing was slower under acidic conditions, yet resulted in an overall bactericidal activity in both conditions in TK assay.

This study also contributes to characterizing the resistance patterns of *E. faecalis* against DLX. Enterococci are intrinsically less susceptible to FQs due to the lower affinity of the DNA gyrase for these antibiotics or the presence of *qnr*-like genes, encoding a protein that protects DNA gyrase from interaction with FQs (25, 26). In this work, several features were highlighted. Although FSMS and MPC values differed among the tested isolates, LVX and DLX appear less likely to promote the selection of *E. faecalis* mutants than MOX, as characterized by lower median FSMS and SI ranges, suggesting that resistance may be more frequently observed with MOX than with LVX or DLX.

From a genetic point of view, mutations at position S84 of GyrA (S83 in *E. coli* numbering) appear to be a key driver of significant increases in DLX MICs in *E. faecalis*. Among mutants harboring newly acquired mutations within the QRDRs (13/19, 68%), substitutions at GyrA position S84 were observed in 11 (85%). Less frequent substitutions were also identified at GyrA position E88 and GyrB position S442. Notably, mutants carrying QRDR mutations in GyrA or GyrB were selected following exposure to MOX (8/13, 62%) or DLX (5/13, 38%), but never on LVX-containing agar plates. Importantly, several mutants with only a single GyrA mutation exhibited MICs above 0.25 mg/L (the FDA breakpoint), suggesting that GyrA alteration alone may be sufficient to confer DLX resistance in *E. faecalis*. Nevertheless, the highest MIC values (4 mg/L) were observed in mutants carrying three different mutations within QRDRs of GyrA, GyrB, and ParC, indicating that multiple mutations may further increase resistance to the molecule. The S84 residue is a well-known hotspot for FQ resistance in multiple bacterial species (19, 27, 28). In general, FQs preferentially target the DNA gyrase in gram-negative bacteria and the topoisomerase IV in gram-positive bacteria, although this can vary depending on the molecules used and the bacterial species (18). DLX achieved an atypical behavior characterized by a nearly equivalent inhibition of both DNA gyrase and topoisomerase IV (15). For example, in *Staphylococcus aureus*, it may even exhibit a slightly higher potency against DNA gyrase, whereas in *Streptococcus pneumoniae*, DNA cleavage mediated by DLX was more efficient for topoisomerase IV than DNA gyrase (15, 29). In this study, most DLX-selected mutants carried GyrA substitutions, whereas mutations in topoisomerase IV subunits were not selected by DLX. In contrast, one mutant harboring a ParE substitution was selected by LVX. These findings suggest that DNA gyrase is the primary target of DLX in *E. faecalis* under these specific experimental conditions, rather than implying a lack of topoisomerase IV inhibition.

A first limitation of this study is the limited number of mutants analyzed and the bias introduced by their selection; consequently, these findings require further investigation to better characterize the target profile of FQs in enterococci. Furthermore, our collection only included a few numbers of FQ-resistant *E. faecalis* strains (*n* = 12), and no *E. faecium* were susceptible to NFX. Despite providing interesting insights, MPC and FSMS interpretation can be challenging as inter-experimental variability is described (21). Finally, although QRDR mutations were screened in this study, additional mechanisms contributing to FQ resistance, such as efflux pump activity, were not investigated, paving the way for further exploration.

Overall, DLX shows a better *in vitro* activity under both neutral and acidic conditions compared to LVX and MOX. In addition, as MIC values decrease under acidic conditions, DLX urinary tract concentration could therefore be greatly above the MIC and potentially above the MPC values, thereby enhancing antibacterial activity and potentially limiting the *in vivo* selection of resistant mutants. It is important to note that based on the FDA breakpoint, DLX activity retains an *in vitro* activity against some FQ-resistant *E. faecalis* isolates (5/12; 42%). However, DLX susceptibility testing should be performed individually for each isolate. In conclusion, DLX appears to be a decent alternative for the treatment of male UTIs caused by FQ-susceptible *E. faecalis* isolates knowing that clinical trials are needed.

## MATERIALS AND METHODS

### Bacterial strains collection

This study included 86 *E. faecalis* and 24 *E. faecium* unrelated clinical isolates responsible for male UTIs, collected between January and December 2021 from Rennes University Hospital (*n* = 83; 68 *E. faecalis* and 15 *E. faecium*) and from other representative French hospitals (*n* = 27; 18 *E. faecalis* and nine *E. faecium*) through the French National Reference Center for Enterococci. Male UTIs were biologically defined as a significant bacteriuria (≥10^3^ CFU/mL) and leukocyturia (≥10^4^/mL) (4). A disk of 10 µg of NFX was used to screen for FQ resistance. All isolates were identified by MALDI-TOF mass spectrometry (MALDI Biotyper Sirius system, Bruker Daltonics, Bremen, Germany) and were stored at −80°C.

### Antimicrobial susceptibility testing

MIC and MBC values of LVX, MOX, and DLX were determined using the BMD reference method in CA-MHB, according to 2025 EUCAST guidelines (https://eucast.org/). For clinical categorization, 2026 CLSI breakpoints were used for LVX (resistant if >4 mg/L), while 2026 FDA breakpoints were used for MOX and DLX (resistant if >2 or >0.25 mg/L, respectively) (30, 31), while ensuring all method guidelines rigorously adhered to EUCAST 2025 standards. MBC values were defined as a 99.9% reduction in bacterial count from baseline, and bactericidal activity was defined as an MBC/MIC ratio ≤4. In addition, 12 *E. faecalis* were selected to represent the overall distribution of DLX MICs and were used for MIC and MBC determination in aCA-MHB. Briefly, aCA-MHB was prepared by adjusting the pH of CA-MHB from 7.2 ± 0.5 to 6 and 5 (± 0.5), respectively, using 37% hydrochloric acid. In CA-MHB, drug concentrations in BMD ranged from 0.004 to 8 mg/L for LVX and MOX and from 0.001 to 2 mg/L for DLX. In aCA-MHB, ranges were from 0.008 to 16 mg/L for both LVX and MOX. Because several strains exhibited lower MICs for DLX, microdilution plates were strain-adapted, covering a broader range of 0.00006 to 2 mg/L for DLX. MBC determination for LVX and MOX was not performed in NFX-resistant strains, as they exhibited high-level resistance to both agents. *E. coli* ATCC 25922, *S. aureus* ATCC 29213, and *E. faecalis* ATCC 29212 were used as QC strains in CA-MHB. All experiments were assessed in triplicates.

### Time-kill assays

DLX *in vitro* bactericidal activity under neutral and acidic conditions was studied through TK assays in BHI at pH 7 and 5 (± 0.5). DLX was used at a concentration of 50-fold the MIC for six selected strains, for which MIC and acidic MIC had previously been determined by BMD in CA-MHB and aCA-MHB, respectively. nBHI was buffered to pH 7 (± 0.5) using 10 M NaOH and 50 mM of 3-(N-morpholino)propanesulfonic acid (MOPS). aBHI was adjusted and buffered to pH 5 (± 0.5) using 37% hydrochloric acid and 50 mM of 4-morpholineethanesulfonic acid (MES). TK assays were performed in 10 mL of nBHI and aBHI with an initial inoculum of ~10^6^–10^7^ CFU/mL after overnight culture in nBHI. Tubes were incubated for 2 h at 35 ± 2°C to ensure that isolates were in mid-exponential phase, then DLX was added to the 10 mL broth. Samples were taken over at 0 (after 2 h of incubation and immediately before the addition of DLX), 3, 6, and 24 h, and colony counts were enumerated by serial dilutions and plotted to generate the TK curves. To avoid carry-over effect, 500 μL of culture was centrifuged, and bacterial pellet was washed with 500 μL of 0.9% NaCl at 3 and 6 h. At 24 h, 1 mL of bacterial culture was centrifuged, washed with 1 mL of 0.9% NaCl, then a second centrifugation was performed, and the final bacterial pellet was washed with 100 μL of 0.9% NaCl in order to concentrate the inoculum 10 times. All serial dilutions were performed on those washed bacterial pellets. Bactericidal activity was defined as a ≥3-log_10_ CFU/mL reduction in bacterial count from baseline at 24 h. TK curves were generated using GraphPad Prism v.9.5.0 software (GraphPad Software, San Diego, CA, USA), and comparisons of bacterial reduction at 24 h between pH 7 and pH 5 were performed using a paired *t*-test on log_10_-transformed T24/T0 ratios.

### Selection of spontaneous mutants, mutant prevention concentration, and frequency of spontaneous mutant selection

Eight *E. faecalis* parental strains were used to generate spontaneous mutants for WGS; but only six of them were retained for MPC and FSMS determination, as high-level FQ-resistant parental strains were excluded. Bacterial cultures were adjusted to a high-density inoculum (~10^10^ CFU/mL), and then 100 μL (~10^9^ CFU) were plated onto BHI agar plates containing LVX, MOX, or DLX at increasing concentrations (2-, 4-, 8-, and 16-fold the MIC of the parental strain). Plates were incubated at 35 ± 2°C for 72 h. For every plate showing growth, suspected mutants were collected and transferred to agar plates with the same compound concentration. Plates were then incubated at 35 ± 2°C for 24 h; if no growth was observed, incubation was extended up to 48 h. Only regrowing colonies were considered suspected spontaneous mutants and stored at −80°C for WGS and MIC testing after regrowth on antibiotic-free media. Given the large number of suspected spontaneous mutants generated, MICs were first determined in a single replicate for screening and then performed in triplicate for isolates selected for WGS. Drug concentrations for BMD ranged from 0.125 to 128 mg/L for LVX, 0.06 to 64 mg/L for MOX, and from 0.008 to 8 mg/L for DLX. Spontaneous resistant mutants were defined as isolates showing a significant increase in MICs (>1 two-fold dilution) of the FQ used for selection compared with the parental strain. The MPC was defined as the minimal concentration of antibiotics that completely inhibited bacterial growth on agar plates after 72 h. As a dense lawn was obtained for several strains at 2-, 4-, and 8-fold the MIC, a second set of experiments was subsequently performed, using a lower inoculum to enable FSMS calculation. Briefly, cultures were adjusted to approximately 10^8^ CFU/mL, then 100 μL (~10^7^ CFU) were plated onto BHI agar to determine FSMS at 2-, 4-, and 8-fold the MIC, following the same protocols as described above and performed at least in duplicate. FSMS was calculated as the number of spontaneous mutants divided by the inoculum plated on agar plates (i.e., 10^7^ or 10^9^), and the highest value obtained from the two inocula was retained. The median FSMS values were calculated from these maximum values. If no growth was visible, FSMS was determined to be lower than the calculated limit of detection (i.e., <1 × 10^−9^).

### Whole-genome sequencing

Genomic DNA of parental strains and a selection of spontaneous mutants was extracted using Quick-DNA Fungal/Bacterial Miniprep kit (Zymo Research, Irvine, California, USA). Quantity of DNA extracted was measured using Nanodrop (Thermo Fisher Scientific, Hampshire, United Kingdom). WGS was performed at BGI platform (Beijing, China). Multi-locus sequence typing (MLST), genome assembly, and genomic comparison between parental strains and related mutants were achieved using Qiagen CLC Genomics Workbench v24.0.1 (Qiagen, Hilden, Germany). Annotation was generated using Prokka tool (http://www.usegalaxy.org). A cut-off of 100 reads for coverage and ≥80% for frequency was used for single nucleotide variant (SNV) analysis. QRDRs of parental isolates were compared with sequences of *E. faecalis* ATCC 29212 reference strain (NZ_CP008816.1). QRDRs were defined as follows (*E. coli* numbering): region between residues 67–106, 426–447, 64–103, and 420–441 for GyrA, GyrB, ParC, and ParE, respectively (32–34). All mutations identified in the QRDRs for both parental and mutant strains were confirmed using specific primers (Table S8), and amplicons were sequenced using Microsynth AG (Balgach, Switzerland).

## Data Availability

The genomic sequences of the parental clinical isolates and derived *in vitro* resistant mutants were deposited in GenBank under BioProject accession number PRJNA1440434.

## SUPPLEMENTAL MATERIAL

Table S1 spectrum.01170-26-s0001.pdf MIC, MBC values, and MBC/MIC ratios of LVX, MOX, and DLX against *E. faecalis* clinical isolates.

Table S2 spectrum.01170-26-s0002.pdf MIC, MBC values, and MBC/MIC ratios of LVX, MOX, and DLX against *E. faecium* clinical isolates.

Table S3 spectrum.01170-26-s0003.pdf MIC values (mg/L) for 12 *E. faecalis* under different pH conditions.

Table S4 spectrum.01170-26-s0004.pdf MBC/MIC ratio of delafloxacin for 12 *E. faecalis* under different pH conditions.

Table S5 spectrum.01170-26-s0005.pdf Summary of time-kill results for six *E. faecalis* at 3, 6, and 24 h for DLX at 50-fold the MIC in pH = 7.2 and 5.

Table S6 spectrum.01170-26-s0006.pdf Range of MIC values (mg/L) related to the number of mutations of *in vitro*-selected mutants.

Table S7 spectrum.01170-26-s0007.pdf Genomic characterization of *in vitro* selected mutant to fluoroquinolones.

Table S8 spectrum.01170-26-s0008.pdf Oligonucleotide primers used in the study.

ASM does not own the copyrights to Supplemental Material that may be linked to, or accessed through, an article. The authors have granted ASM a non-exclusive, world-wide license to publish the Supplemental Material files. Please contact the corresponding author directly for reuse.
