# Supplementary material for: In vitro activity of delafloxacin against enterococcal isolates from male urinary tract infections and genetic determinants of resistance
Source: Microbiol Spectr. 2026 Aug 24;14(10):e01170-26. doi: 10.1128/spectrum.01170-26 (PMC13689276; doi:10.1128/spectrum.01170-26)
Supplement: Supplementary file 1 [file spectrum.01170-26-s0001.pdf]

TABLE S1 MIC, MBC values and MBC/MIC ratios of LVX, MOX and DLX against *E. faecalis* clinical isolates

Strains	Species	NFX	LVX			MOX			DLX		
			MIC (mg/L)	MBC (mg/L)	MBC/MIC	MIC (mg/L)	MBC (mg/L)	MBC/MIC	MIC (mg/L)	MBC (mg/L)	MBC/MIC
21-113_ENFA1	*E. faecalis*	S	2	2	1	0.5	0.5	1	0.125	0.125	1
21-316_ENFA1	*E. faecalis*	S	1	1	1	0.25	0.25	1	0.06	0.125	2
21-367_ENFA1	*E. faecalis*	S	1	4	4	0.25	0.25	1	0.06	0.06	1
21-374_ENFA1	*E. faecalis*	S	1	1	1	0.25	0.25	1	0.06	0.06	1
21-412_ENFA1	*E. faecalis*	S	1	1	1	0.125	0.25	2	0.06	0.06	1
21-544_ENFA1	*E. faecalis*	S	1	2	2	0.25	0.5	2	0.06	0.125	2
21-562_ENFA1	*E. faecalis*	S	2	2	1	0.5	0.5	1	0.125	0.25	2
21-644_ENFA1	*E. faecalis*	S	1	2	2	0.25	0.25	1	0.06	0.125	2
ENFA1_001	*E. faecalis*	S	2	8	4	0.5	1	2	0.125	0.5	4
ENFA1_002	*E. faecalis*	S	1	2	2	0.25	0.5	2	0.06	0.125	2
ENFA1_003	*E. faecalis*	S	2	2	1	0.5	0.5	1	0.125	0.25	2
ENFA1_004	*E. faecalis*	S	1	1	1	0.125	0.25	2	0.03	0.06	2
ENFA1_005	*E. faecalis*	S	2	4	2	0.5	0.5	1	0.125	0.25	2
ENFA1_006	*E. faecalis*	S	1	1	1	0.25	0.25	1	0.06	0.06	1
ENFA1_007	*E. faecalis*	S	1	1	1	0.25	0.25	1	0.06	0.125	2
ENFA1_008	*E. faecalis*	S	1	1	1	0.25	0.25	1	0.06	0.06	1
ENFA1_009	*E. faecalis*	S	1	2	2	0.5	0.5	1	0.06	0.125	2
ENFA1_010	*E. faecalis*	S	1	1	1	0.25	0.25	1	0.03	0.06	2
ENFA1_011	*E. faecalis*	S	1	2	2	0.25	0.5	2	0.06	0.125	2
ENFA1_012	*E. faecalis*	S	1	2	2	0.25	0.5	2	0.06	0.125	2
ENFA1_013	*E. faecalis*	S	1	8	8	0.25	0.25	1	0.125	0.125	1
ENFA1_014	*E. faecalis*	S	1	>8	>8	0.25	1	4	0.06	0.125	2
ENFA1_015	*E. faecalis*	S	1	1	1	0.25	0.25	1	0.06	0.125	2
ENFA1_018	*E. faecalis*	S	0.5	1	2	0.125	0.125	1	0.03	0.03	1
ENFA1_019	*E. faecalis*	S	0.5	1	2	0.125	0.125	1	0.03	0.03	1
ENFA1_020	*E. faecalis*	S	1	1	1	0.25	0.25	1	0.125	0.125	1
ENFA1_021	*E. faecalis*	S	1	1	1	0.125	0.125	1	0.06	0.06	1
ENFA1_022	*E. faecalis*	S	2	2	1	0.5	0.5	1	0.125	0.125	1
ENFA1_023	*E. faecalis*	S	1	1	1	0.125	0.25	2	0.06	0.06	1
ENFA1_024	*E. faecalis*	S	1	1	1	0.25	0.25	1	0.06	0.06	1
ENFA1_025	*E. faecalis*	S	1	1	1	0.25	0.25	1	0.06	0.06	1
ENFA1_026	*E. faecalis*	S	1	2	2	0.25	0.5	2	0.06	0.125	2
ENFA1_027	*E. faecalis*	S	1	2	2	0.25	0.25	1	0.06	0.125	2
ENFA1_028	*E. faecalis*	S	1	1	1	0.25	0.25	1	0.06	0.06	1
ENFA1_029	*E. faecalis*	S	1	2	2	0.25	0.5	2	0.125	0.125	1
ENFA1_030	*E. faecalis*	S	1	1	1	0.25	0.25	1	0.06	0.125	2
ENFA1_032	*E. faecalis*	S	1	1	1	0.25	0.25	1	0.06	0.06	1
ENFA1_033	*E. faecalis*	S	1	2	2	0.25	0.5	2	0.06	0.125	2
ENFA1_034	*E. faecalis*	S	1	1	1	0.25	0.25	1	0.06	0.06	1
ENFA1_035	*E. faecalis*	S	1	1	1	0.125	0.25	2	0.03	0.06	2
ENFA1_037	*E. faecalis*	S	1	1	1	0.25	0.25	1	0.06	0.06	1
ENFA1_038	*E. faecalis*	S	1	2	2	0.25	0.25	1	0.125	0.125	1

–, not determined; DLX, delafloxacin; LVX, levofloxacin; MOX, moxifloxacin; R, resistant; S, susceptible.

TABLE S1 *Cont.*

Strains	Species	NFX	LVX			MOX			DLX		
			MIC (mg/L)	MBC (mg/L)	MBC/MIC	MIC (mg/L)	MBC (mg/L)	MBC/MIC	MIC (mg/L)	MBC (mg/L)	MBC/MIC
ENFA1_039	*E. faecalis*	S	1	1	1	0.125	0.25	2	0.03	0.06	2
ENFA1_040	*E. faecalis*	S	1	2	2	0.25	0.5	2	0.06	0.125	2
ENFA1_041	*E. faecalis*	S	1	2	2	0.25	0.5	2	0.125	0.125	1
ENFA1_043	*E. faecalis*	S	0.5	1	2	0.125	0.25	2	0.03	0.06	2
ENFA1_044	*E. faecalis*	S	1	1	1	0.25	0.25	1	0.06	0.125	2
ENFA1_045	*E. faecalis*	S	1	2	2	0.25	0.5	2	0.06	0.125	2
ENFA1_046	*E. faecalis*	S	2	2	1	0.25	0.5	2	0.125	0.125	1
ENFA1_047	*E. faecalis*	S	1	1	1	0.25	0.25	1	0.06	0.06	1
ENFA1_048	*E. faecalis*	S	1	1	1	0.25	0.25	1	0.06	0.125	2
ENFA1_050	*E. faecalis*	S	1	2	2	0.25	0.5	2	0.06	0.125	2
ENFA1_051	*E. faecalis*	S	1	> 8	> 8	0.25	2	8	0.06	1	16
ENFA1_052	*E. faecalis*	S	1	1	1	0.25	0.25	1	0.06	0.06	1
ENFA1_053	*E. faecalis*	S	1	1	1	0.25	0.25	1	0.06	0.06	1
ENFA1_054	*E. faecalis*	S	1	1	1	0.25	0.25	1	0.06	0.06	1
ENFA1_055	*E. faecalis*	S	2	2	1	0.25	0.5	2	0.06	0.125	2
ENFA1_056	*E. faecalis*	S	0.5	2	4	0.125	1	8	0.03	0.25	8
ENFA1_057	*E. faecalis*	S	0.5	1	2	0.125	2	16	0.03	0.06	2
ENFA1_058	*E. faecalis*	S	1	1	1	0.25	0.25	1	0.125	0.125	1
ENFA1_059	*E. faecalis*	S	1	2	2	0.25	0.25	1	0.06	0.06	1
ENFA1_060	*E. faecalis*	S	1	1	1	0.25	0.5	2	0.06	0.125	2
ENFA1_061	*E. faecalis*	S	1	1	1	0.125	0.25	2	0.03	0.06	2
ENFA1_062	*E. faecalis*	S	1	1	1	0.125	0.25	2	0.03	0.03	1
ENFA1_063	*E. faecalis*	S	1	1	1	0.25	0.25	1	0.06	0.06	1
ENFA1_064	*E. faecalis*	S	1	1	1	0.25	0.25	1	0.03	0.06	2
ENFA1_065	*E. faecalis*	S	1	1	1	0.25	0.25	1	0.06	0.125	2
ENFA1_066	*E. faecalis*	S	1	1	1	0.25	0.25	1	0.03	0.06	2
ENFA1_067	*E. faecalis*	S	2	2	1	0.5	0.5	1	0.125	0.125	1
ENFA1_069	*E. faecalis*	S	2	2	1	0.25	0.5	2	0.06	0.125	2
ENFA1_071	*E. faecalis*	S	1	2	2	0.25	0.25	1	0.06	0.125	2
ENFA1_072	*E. faecalis*	S	1	1	1	0.125	0.25	2	0.06	0.06	1
ENFA1_073	*E. faecalis*	S	1	2	2	0.25	0.5	2	0.06	0.125	2
ENFA1_074	*E. faecalis*	S	2	2	1	0.5	0.5	1	0.125	0.25	2
21-247_ENFA1	*E. faecalis*	R	> 8	–	–	> 8	–	–	1	2	2
21-439_ENFA1	*E. faecalis*	R	> 8	–	–	8	–	–	0.5	0.5	1
21-450_ENFA1	*E. faecalis*	R	> 8	–	–	8	–	–	0.25	0.5	2
21-517_ENFA1	*E. faecalis*	R	> 8	–	–	2	–	–	0.125	0.125	1
21-535_ENFA1	*E. faecalis*	R	> 8	–	–	8	–	–	0.25	0.5	2
21-597_ENFA1	*E. faecalis*	R	> 8	–	–	> 8	–	–	1	2	2
21-636_ENFA1	*E. faecalis*	R	> 8	–	–	> 8	–	–	1	2	2
21-638_ENFA1	*E. faecalis*	R	> 8	–	–	> 8	–	–	1	2	2
21-675_ENFA1	*E. faecalis*	R	> 8	–	–	8	–	–	0.25	1	4
21-726_ENFA1	*E. faecalis*	R	> 8	–	–	> 8	–	–	0.5	0.5	1
ENFA1_031	*E. faecalis*	R	> 8	–	–	8	–	–	0.25	0.5	2
ENFA1_042	*E. faecalis*	R	> 8	–	–	> 8	–	–	1	2	2

–, not determined; DLX, delafloxacin; LVX, levofloxacin; MOX, moxifloxacin; R, resistant; S, susceptible.
