# Supplementary material for: In vitro activity of delafloxacin against enterococcal isolates from male urinary tract infections and genetic determinants of resistance
Source: Microbiol Spectr. 2026 Aug 24;14(10):e01170-26. doi: 10.1128/spectrum.01170-26 (PMC13689276; doi:10.1128/spectrum.01170-26)
Supplement: Supplementary file 2 [file spectrum.01170-26-s0002.pdf]

TABLE S2 MIC, MBC values and MBC/MIC ratios of LVX, MOX and DLX against *E. faecium* clinical isolates

			LVX			MOX			DLX		
Strains	Species	NFX	MIC (mg/L)	MBC (mg/L)	MBC/MIC	MIC (mg/L)	MBC (mg/L)	MBC/MIC	MIC (mg/L)	MBC (mg/L)	MBC/MIC
21-031_ENFA2	*E. faecium*	R	> 8	–	–	> 8	–	–	> 8	–	–
21-061_ENFA2	*E. faecium*	R	> 8	–	–	> 8	–	–	> 8	–	–
21-495_ENFA2	*E. faecium*	R	> 8	–	–	> 8	–	–	> 8	–	–
21-497_ENFA2	*E. faecium*	R	> 8	–	–	> 8	–	–	> 8	–	–
21-521_ENFA2	*E. faecium*	R	> 8	–	–	> 8	–	–	> 8	–	–
21-555_ENFA2	*E. faecium*	R	> 8	–	–	> 8	–	–	> 8	–	–
21-574_ENFA2	*E. faecium*	R	> 8	–	–	> 8	–	–	> 8	–	–
21-583_ENFA2	*E. faecium*	R	> 8	–	–	> 8	–	–	8	–	–
21-737_ENFA2	*E. faecium*	R	> 8	–	–	> 8	–	–	> 8	–	–
ENFA2_001	*E. faecium*	R	> 8	–	–	> 8	–	–	> 8	–	–
ENFA2_002	*E. faecium*	R	> 8	–	–	> 8	–	–	> 8	–	–
ENFA2_003	*E. faecium*	R	> 8	–	–	> 8	–	–	> 8	–	–
ENFA2_004	*E. faecium*	R	> 8	–	–	> 8	–	–	> 8	–	–
ENFA2_005	*E. faecium*	R	> 8	–	–	> 8	–	–	> 8	–	–
ENFA2_006	*E. faecium*	R	> 8	–	–	> 8	–	–	> 8	–	–
ENFA2_007	*E. faecium*	R	> 8	–	–	> 8	–	–	> 8	–	–
ENFA2_008	*E. faecium*	R	> 8	–	–	> 8	–	–	> 8	–	–
ENFA2_009	*E. faecium*	R	> 8	–	–	> 8	–	–	8	–	–
ENFA2_011	*E. faecium*	R	> 8	–	–	> 8	–	–	> 8	–	–
ENFA2_013	*E. faecium*	R	> 8	–	–	> 8	–	–	> 8	–	–
ENFA2_016	*E. faecium*	R	> 8	–	–	> 8	–	–	> 8	–	–
ENFA2_017	*E. faecium*	R	> 8	–	–	> 8	–	–	> 8	–	–
ENFA2_018	*E. faecium*	R	> 8	–	–	> 8	–	–	> 8	–	–
ENFA2_020	*E. faecium*	R	> 8	–	–	8	–	–	4	–	–

–, not determined; DLX, delafloxacin; LVX, levofloxacin; MOX, moxifloxacin; R, resistant; S, susceptible.
