# Supplementary material for: In vitro activity of delafloxacin against enterococcal isolates from male urinary tract infections and genetic determinants of resistance
Source: Microbiol Spectr. 2026 Aug 24;14(10):e01170-26. doi: 10.1128/spectrum.01170-26 (PMC13689276; doi:10.1128/spectrum.01170-26)
Supplement: Supplementary file 3 [file spectrum.01170-26-s0003.pdf]

TABLE S3 MIC values (mg/L) for 12 *E. faecalis* under different pH conditions

	LVX			MOX			DLX		
Strains	pH= 7.2	pH= 6	pH= 5	pH= 7.2	pH= 6	pH= 5	pH= 7.2	pH= 6	pH= 5
21-412_ENFA1	1	2	8	0.125	0.5	4	0.06	0.016	0.008
21-562_ENFA1	2	4	16	0.5	1	4	0.125	0.016	0.008
21-636_ENFA1	> 8	> 16	> 16	> 8	> 16	> 16	1	0.25	0.25
ENFA1_001	2	4	16	0.5	1	4	0.125	0.016	0.008
ENFA1_002	1	4	16	0.25	1	4	0.06	0.016	0.016
ENFA1_018	0.5	2	4	0.125	0.5	1	0.03	0.004	0.001
ENFA1_019	0.5	2	4	0.125	0.25	1	0.03	0.004	0.001
ENFA1_030	1	2	8	0.25	1	4	0.06	0.016	0.008
ENFA1_031	> 8	> 16	> 16	8	> 16	> 16	0.25	0.125	0.06
ENFA1_039	1	2	8	0.125	0.5	1	0.03	0.016	0.004
ENFA1_042	> 8	> 16	> 16	> 8	> 16	> 16	1	0.125	0.25
ENFA1_069	2	4	16	0.25	1	4	0.06	0.016	0.004

DLX, delafloxacin; LVX, levofloxacin; MOX, moxifloxacin.
