# Supplementary material for: In vitro activity of delafloxacin against enterococcal isolates from male urinary tract infections and genetic determinants of resistance
Source: Microbiol Spectr. 2026 Aug 24;14(10):e01170-26. doi: 10.1128/spectrum.01170-26 (PMC13689276; doi:10.1128/spectrum.01170-26)
Supplement: Supplementary file 4 [file spectrum.01170-26-s0004.pdf]

TABLE S4 MBC/MIC ratio of delafloxacin for 12 *E. faecalis* in different pH conditions

	MBC/MIC		
Strains	pH= 7.2	pH= 6	pH= 5
21-412_ENFA1	1	1	1
21-562_ENFA1	2	2	2
21-636_ENFA1	2	> 8	> 8
ENFA1_001	4	2	8
ENFA1_002	2	8	8
ENFA1_018	1	4	> 64
ENFA1_019	1	4	> 64
ENFA1_030	2	2	2
ENFA1_031	2	1	2
ENFA1_039	2	2	64
ENFA1_042	2	2	> 8
ENFA1_069	2	1	4
