# Supplementary material for: In vitro activity of delafloxacin against enterococcal isolates from male urinary tract infections and genetic determinants of resistance
Source: Microbiol Spectr. 2026 Aug 24;14(10):e01170-26. doi: 10.1128/spectrum.01170-26 (PMC13689276; doi:10.1128/spectrum.01170-26)
Supplement: Supplementary file 5 [file spectrum.01170-26-s0005.pdf]

TABLE S5 Summary of time-kill results for 6 *E. faecalis* at 3, 6, and 24h for DLX at 50-fold the MIC in pH= 7.2 and 5[a]

Strains	Condition	3h	6h	24h
21-412_ENFA1	pH= 7.2	**-3.06**	**-3.06**	**-4.17**
	pH= 5	-1.79	-2.41	**-3.81**
21-562_ENFA1	pH= 7.2	-1.95	-2.92	**-5.29**
	pH= 5	-1.25	-1.91	-2.92
21-636_ENFA1	pH= 7.2	-2.65	**-3.85**	**-4.94**
	pH= 5	-0.60	-1.30	-1.40
ENFA1_018	pH= 7.2	-2.93	**-3.40**	**-5.24**
	pH= 5	-0.81	-1.37	**-3.03**
ENFA1_019	pH= 7.2	**-3.13**	**-3.94**	**-4.64**
	pH= 5	-1.27	-1.79	**-3.83**
ENFA1_039	pH= 7.2	-2.75	**-3.67**	**-5.06**
	pH= 5	-1.61	-2.26	**-4.75**

[a]Results are expressed as the mean difference in bacterial counts (log_{10} CFU/mL) from the baseline inoculum.

Bactericidal activity, defined as a decrease of ≥3 log_{10} CFU/mL at 24 h compared with the initial inoculum is indicated in bold and underlined.
