# Supplementary material for: In vitro activity of delafloxacin against enterococcal isolates from male urinary tract infections and genetic determinants of resistance
Source: Microbiol Spectr. 2026 Aug 24;14(10):e01170-26. doi: 10.1128/spectrum.01170-26 (PMC13689276; doi:10.1128/spectrum.01170-26)
Supplement: Supplementary file 6 [file spectrum.01170-26-s0006.pdf]

TABLE S6 Range of MIC values (mg/L) related to the number of mutations of in vitro selected mutants

No. of isolates	QRDR					MIC range (mg/L)		
	GyrA	GyrB	ParC	ParE	Total mutations	LVX	MOX	DLX
6	–	–	–	–	0	1 – 8	0.25 - 2	0.125 - 1
9	1	–	–	–	1	1 - 4	0.5 - 4	0.125 - 1
1	–	1	–	–	1	2	1	0.06
1	–	–	–	1	1	4	1	0.06
4	2	–	1	–	3	32 - 64	8 - 32	0.25 - 4
1	1	1	1	–	3	64	32	4
