# Supplementary material for: In vitro activity of delafloxacin against enterococcal isolates from male urinary tract infections and genetic determinants of resistance
Source: Microbiol Spectr. 2026 Aug 24;14(10):e01170-26. doi: 10.1128/spectrum.01170-26 (PMC13689276; doi:10.1128/spectrum.01170-26)
Supplement: Supplementary file 7 [file spectrum.01170-26-s0007.pdf]

TABLE S7 Genomic characterization of in vitro selected mutant to fluoroquinolones

Strain	Isolation	MIC (mg/L)			QRDR	Other mutations[a]		
		LVX	MOX	DLX	mutations[a]	Gene	Protein	Mutation
ENFA1_019		0.5	0.125	0.03	0			
Mutant 1	DLX	1	0.25	0.125	0	*metP*<br>*uvrA*<br>–<br>–	Methionine ABC transporter permease<br>Excinuclease ABC subunit UvrA<br>Isopeptide-forming domain-containing fimbrial protein<br>Intergenic region	Gly42Glu<br>His862Gln<br>Val912Phe<br>–
Mutant 2	DLX	2	1	0.25	1	cshA<br>–<br>–	Degradosome RNA helicase<br>dNTPase<br>Intergenic region	Ala514fs<br>Val173Leu<br>–
Mutant 3	MOX	2	1	0.06	1	*rplB*<br>–	50S ribosomal protein L2<br>Intergenic region	His230Gln<br>–
ENFA1_006		1	0.25	0.06	0			
Mutant 1	DLX	4	1	0.5	0	*eno*	Phosphopyruvate hydratase	Asn148Asp
Mutant 2	DLX	8	2	0.5	0	*eno*	Phosphopyruvate hydratase	Asn148Asp
Mutant 3	LVX	4	0.5	0.25	0	*infB*	Translation initiation factor IF-2	Glu200*
Mutant 4	MOX	4	1	0.25	1	*guaA*	Glutamine-hydrolyzing GMP synthase	Pro428fs
ENFA1_004		1	0.125	0.03	0			
Mutant 1	DLX	2	1	0.25	1	*yhdG/yhfP*	Quinone oxidoreductase	Glu76Gly
Mutant 2	LVX	2	0.25	0.125	0	*yhdG/yhfP*	Quinone oxidoreductase	Glu76Gly
Mutant 3	LVX	4	1	0.06	1	*rplW*<br>–<br>*yhdG/yhfP*<br>*erfD*	50S ribosomal protein L23<br>ABC transporter ATP-binding protein<br>Quinone oxidoreductase<br>Multidrug ABC transporter	Tyr68fs<br>Glu97Lys<br>Glu76Gly<br>Leu352fs
Mutant 4	MOX	1	0.5	0.125	1	*yhdG/yhfP*	Quinone oxidoreductase	Glu76Gly
Mutant 5	MOX	4	2	0.25	1	*yhdG/yhfP*	Quinone oxidoreductase	Glu76Gly
ENFA1_003		2	0.5	0.125	0			
Mutant 1	DLX	4	2	1	1	*pyk*<br>*ace*	Pyruvate kinase<br>Collagen binding domain-containing protein	Ala190Val<br>Glu387del
Mutant 2	MOX	4	4	0.5	1	–<br>*uvrB*<br>*ace*<br>–	L-threonylcarbamoyladenylate synthase<br>Excinuclease ABC subunit<br>Collagen binding domain-containing protein<br>TPM domain-containing protein	Gly90Val<br>Asp570fs<br>Glu387del<br>Cys354*
ENFA1_002		1	0.25	0.06	0			
Mutant 1	MOX	4	2	1	1	*gndA*<br>*cydC*<br>–	NADP-dependent phosphogluconate dehydrogenase<br>Thiol reductant ABC exporter subunit<br>Adenylosuccinate synthase	Leu354fs<br>Ala285Val<br>Ala5fs
Mutant 2	MOX	4	2	1	1	*pfkB*	1-phosphofructokinase	Gly164_Met168del
21562		2	0.5	0.125	0			
Mutant 1	LVX	4	2	1	0	*eno*	Phosphopyruvate hydratase	Ala244Thr
21517		16	2	0.125	2			
Mutant 1	DLX	64	32	4	3	–	Permease prefix domain 1-containing protein	Cys135fs
Mutant 2	MOX	32	32	0.5	3	–	–	–
Mutant 3	MOX	64	32	4	3	–	Intergenic region	–
Mutant 4	MOX	32	8	0.25	3	*rodA*	Cell cycle protein	Ala57Val
21439		32	8	0.5	2			
Mutant 1	DLX	64	32	4	3	–	–	–

DLX, delafloxacin; LVX, levofloxacin; MOX, moxifloxacin.

[a]Only acquired mutations outside the FQ targets (i.e., GyrA, GyrB, ParC, ParE) are shown.
