# Supplementary material for: In vitro activity of delafloxacin against enterococcal isolates from male urinary tract infections and genetic determinants of resistance
Source: Microbiol Spectr. 2026 Aug 24;14(10):e01170-26. doi: 10.1128/spectrum.01170-26 (PMC13689276; doi:10.1128/spectrum.01170-26)
Supplement: Supplementary file 8 [file spectrum.01170-26-s0008.pdf]

TABLE S8 Oligonucleotide primers used in the study

Primer name	Nucleotide sequence (5'-3')
gyrA-F	AACCTGTTCATCGCCGAATC
gyrA-R	AAGTTTGGAAAACGAGCAGGT
gyrB-F	TGGAAAATCCAACAGTCGGC
gyrB-R	TTCGAATATGCGCACCATCG
parC-F	ATTCAAGAACGGGCCCTACC
parC-R	TTGGCTGGTAAAACAGTGGG
parE-F	CGTCGTAGGCGAACAAATGG
parE-R	CTTGAATATGGGCACCATCGG
